# Suicidal intoxication with mercury chloride

**DOI:** 10.1007/s11419-022-00653-7

**Published:** 2022-12-24

**Authors:** Sławomir Majdanik, Barbara Potocka-Banaś, Sebastian Glowinski, Sylwester Luzny

**Affiliations:** 1 Department of Clinical and Forensic Toxicology, Pomeranian Medical University in Szczecin, Powstanców Wielkopolskich 72, 70-111 Szczecin, Poland; 2 Institute of Physical Culture and Health, State Higher School of Vocational Education in Koszalin, Lesna 1, 75-582 Koszalin, Poland; 3 Department of Mechanical Engineering, Koszalin University of Technology, Sniadeckich 2, 75-453 Koszalin, Poland

**Keywords:** Mercury chloride, Poisoning, Suicide by intoxication

## Abstract

**Purpose:**

Poisoning with elemental metals and metallic compounds was much more frequent in the past, and was related, among other things, to lifestyle and the lack of appropriate toxicological diagnostics. One example is mercury, which is being gradually eliminated but still has many different applications as a pure metal or in the form of various compounds. The paper presents a case of suicidal poisoning with mercury chloride (corrosive sublimate).

**Methods:**

Forensic and toxicological tests including inductively coupled plasma mass spectrometry (ICP-MS) were at the Department of Forensic Medicine, PMU in Szczecin.

**Results:**

The patient before death had a range of symptoms such as epigastric pain, vomiting of the stomach contents, central cyanosis with tachycardia, tremors, severe shortness of breath with wheezing, difficulty swallowing, slurred speech, rales in the lungs, and diarrhea. The concentration of mercury measured by ICP-MS was 191 mg/L for a blood sample collected antemortem, and 147 mg/L for a blood sample collected at autopsy. Both concentrations of mercury are regarded as lethal. The post-mortem examination revealed signs of extensive thrombotic necrosis in some internal organs.

**Conclusions:**

Mercuric chloride has an estimated human fatal dose of between 1 and 4 g. It can produce a range of toxic effects, including corrosive injury, severe gastrointestinal disturbances, acute renal failure, circulatory collapse, and eventual death. The presented case of fatal poisoning with mercury chloride, due to the type of agent used, is now interesting in toxicological practice.

**Supplementary Information:**

The online version contains supplementary material available at 10.1007/s11419-022-00653-7.

## Introduction

Mercury (Hg) is a chemical element classified as a transition metal. Although there is a trend to eliminate mercury in many areas, it is still used in electronic parts, chemical synthesis, the production of measuring equipment, the dyeing industry, and medicine [1].

Currently, exposure to mercury in the general population is associated with the consumption of fish and fish products, while cases of poisoning with vaporized mercury most often result from occupational exposure, primarily in the mining industry or in plants where mercury compounds are used [2, 3]. On the other hand, inorganic mercury compounds are the cause of suicidal or accidental acute poisoning [4].

Metallic Hg injection, often used as a method of suicide, is regularly observed among young people [5–8]. Mercury penetrates the human body through the digestive tract, respiratory tract, and the skin. Organomercury compounds are more easily absorbed from the gastrointestinal tract than inorganic compounds [9, 10]. Most cases of poisoning with inorganic forms of Hg are acute. These are mainly intoxications with mercury (II) chloride (HgCl_2_), commonly known as corrosive sublimate. The mean lethal dose after ingestion is about 1–4 g of HgCl_2_. Symptoms of poisoning include acute gastroenteritis with vomiting and diarrhea, as well as acute renal failure. Cases of chronic poisoning resulting in kidney damage are rare. Blood levels of Hg below 10 μg/L are considered normal. The first symptoms of poisoning may occur when the Hg concentration in the blood is 20 µg/L [11, 12].

The safe concentration of Hg in the body is difficult to determine due to the different toxicities of mercury compounds and their often-delayed effects. Undoubtedly, the toxicity of Hg depends on its chemical form, route of exposure, dose, and time of exposure, as well as the personal characteristics of the exposed person. Mercury does not have any physiological function in the human body, and its harmful effects have been reported many times [10–12].

HgCl_2_ (corrosive sublimate) is a white, crystalline solid substance that has been used in the past as a disinfectant due to its protein denaturing properties. Cases of poisoning with corrosive sublimate are common and acute and are more frequently related with suicide attempts than occupational exposure. About 10% of HgCl_2_ is absorbed in the gastrointestinal tract [4, 11].

Hg levels in biological materials (blood, urine, and hair) are currently determined using instrumental methods, such as inductively coupled plasma mass spectrometry (ICP-MS) or atomic absorption spectrometry (AAS). Increasingly advanced techniques now allow for the detection of very low Hg concentrations [11, 13].

Hg has a critical role in many health problems with harmful consequences, with Hg primarily targeting the brain and its components, such as the central nervous system (CNS). A correlation between Hg and Alzheimer's disease (AD) based on the known literature in the occupational field was presented in [14]. Authors described the interactions between Hg and AD in neuronal degenerations, apoptosis, autophagy, oxidative stress (OS), mitochondrial malfunctions, gastrointestinal (GI) microflora, infertility, and altering gene expression.

## Analytical methodology

A venous blood samples were collected before medical procedures and post-mortem.

Each time, blood samples were collected in standard EDTA/NaF tubes (Vacutainer^®^ System, Becton, Dickinson, USA). Collected blood was stored and transported at 2–8 °C.

Drug screening was performed using liquid chromatography–tandem mass spectrometry. Whole blood samples were deproteinized with acetonitrile, and after mixing and centrifugation, the supernatant was diluted with deionized water and subjected to analysis. Separation was carried out using a Shimadzu Nexera XR (Kyoto, Japan). Mass spectrometer: AB Sciex TripleTOF 5600 + (Framingham, MA, USA), ion source DuoSpray (APCI/ESI) with calibrant delivery system (CDS). The stationary phase was a monolithic column Chromolithic^®^ Performance RP-18e, 100–2 mm (Merck, Darmstadt, Germany). Mobile phase was composed of two solutions: (A) 0.1% formic acid in water and (B) 0.1% formic acid in acetonitrile. The analysis was carried out at a flow rate of 0.4 mL/min. and a gradient slope of 95–5% in 10 min. Analysis of results: software Peak View v. 2.1 2 Master View.

Determination of mercury was performed using ICP-MS PerkinElmer ELAN DRC-e (Waltham, MA, USA). The spectrometer was calibrated using a Multi-Element Calibration Standard 10 mg/L (PerkinElmer) diluted in matching matrix. Rhodium was used as the internal standard, and Oxygen (Messer, Chorzow, Poland) was used as reaction gas. Standard analysis procedure assumes a 30-fold dilution of serum/plasma in blank reagent composed of high purity water, TMAH (AlfaAesar, Germany), Triton X-100 (PerkinElemer), EDTA (Merck), butanol (Merck), Rhodium-105 (PerkinElmer), and gold (VWR, Germany). Unexpected high concentration of that element led to expansion of calibration curve to 4 points—1, 5, 10, and 50 µg/L (from standard 1, 5, 10 µg/L) and required a dilution of samples 10 000 times.

## Case description

The article presents a case of suicidal poisoning with HgCl_2_. As recorded in the case file, a woman (38) worked in a laboratory and had the keys to a safe where a range of poisonous chemical compounds were stored. In her apartment, where the poisoning took place, a package containing HgCl_2_ (corrosive sublimate) was found.

The paramedic team was called to the patient's place of residence at 06:54 a.m. by one of the household members. When paramedics arrived at 07:07 a.m. the patient was attempting to vomit. She said when interviewed that at 06:40 a.m., she had taken mercury chloride in a suicide attempt (the amount of the ingested substance was not specified). She also mentioned that for the previous 3 months, she had been taking medications for depression (escitalopram, trazodone). The physical examination of the conscious patient, who was reporting abdominal pain at that time, did not yet reveal any signs of respiratory or circulatory failure.

The patient was admitted to the Hospital Emergency Department (HED) at 07:34 a.m. (about 1 h after the ingestion of the poison), and then, she reported epigastric pain and vomited stomach contents. At that time, the patient was diagnosed with central cyanosis, tachycardia 110/min, blood pressure 113/78 mmHg, tremors, severe shortness of breath with wheezing, difficulty swallowing, slurred speech, rales in the lungs, abdominal pain, and diarrhea. A blood test revealed a very high hematocrit level, which could be associated with severe dehydration and was a secondary cause for the increase in other parameters (hemoglobin 20 g/dL, HCT 61.6%, RBC 7.11 million/ul, WBC 32,900/ul, PLT 374,000/ul). Due to the very strong hemolysis, biochemical blood tests were abandoned. The concentration of mercury in a sample of blood obtained from the patient was 191 mg/L. The mercury level was measured using ICP-MS.

The patient was rehydrated, treated with levonor, controloc, and fentanyl, intubated and connected to a ventilator. Gastric lavage was not performed. At 10:25 a.m., the patient had a sudden cardiac arrest (SCA) and was pronounced dead after an unsuccessful resuscitation procedure.

**Fig. 1:**
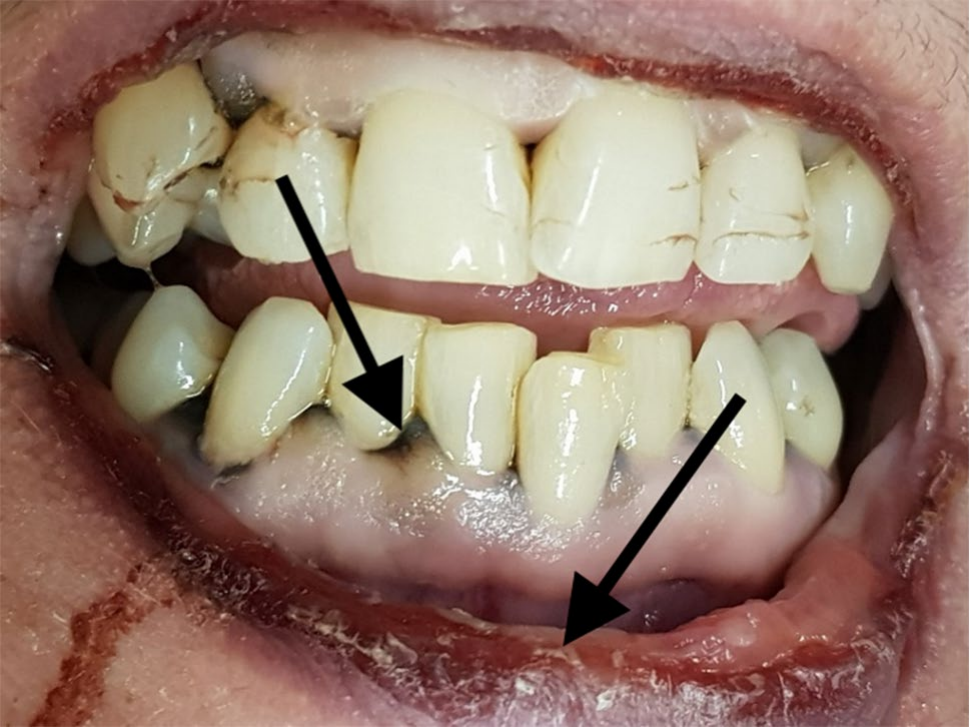
Pathologies on the lips and gums

A prosecutor initiated an investigation into the case and ordered a forensic autopsy. The autopsy revealed significant pathologies, including dark brown discoloration with superficial necrosis of the line between lips and mucosa, and blackish discoloration of the gums at the base of some teeth (Fig. 1).

**Fig. 2:**
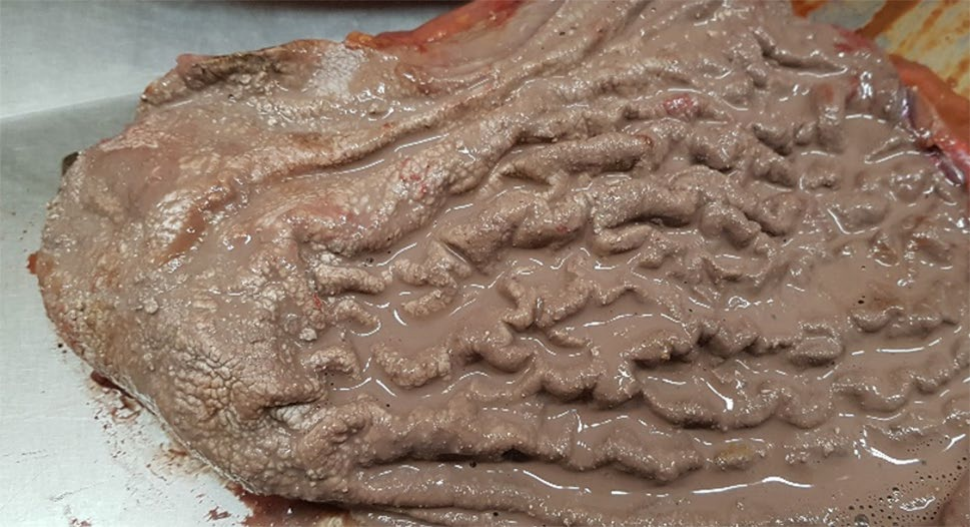
Gastric mucosa

Post-mortem examination also revealed very soft esophageal mucosa and its flaky exfoliation, whitish hard lesions on the entire stomach wall (thrombotic necrosis) with a gray–white discoloration of the rough mucosa, the presence of 50 mL of a beige liquid in the stomach lumen (Fig. 2), very soft duodenal mucosa, as well as watery content of the stomach and watery fecal masses in further segments of the digestive tract.

There were also signs of congestion in internal organs, including cerebral and pulmonary edema, confirmed by further histopathological examination. At the autopsy blood was collected for toxicological tests. Mercury at a concentration of 147 mg/L (ICP-MS) was quantified. Additionally, medications: fentanyl, lidocaine, and midazolam (which were administered during medical procedures), as well as citalopram and trazodone (which the deceased took for the treatment of depression), were detected qualitatively in material preserved for analysis. No other non-volatile organic compounds of exogenous origin with intoxicating, psychotropic, or pharmacological effects were found in the tested material.

Considering the findings from the autopsy and additional tests, suicidal poisoning with mercury chloride was accepted as the cause of death. Consequently, the investigation into this case was dropped by the prosecutor.

## Discussion

Cases of fatal poisoning with inorganic mercury compounds are currently not so often, in comparison to old years [11]. The general discussion of evolutionary aspects of mercury, protective and toxic mechanisms, and ends on a note that mercury is still an “element of mystery” is a current public health concern [15].

In the presented case of poisoning with corrosive sublimate, the patient before death had a range of symptoms such as epigastric pain, vomiting of the stomach contents, central cyanosis with tachycardia, tremors, severe shortness of breath with wheezing, difficulty swallowing, slurred speech, rales in the lungs, and diarrhea. These symptoms were also observed in other cases of poisoning with mercury chloride described in the medical literature [10, 13]. It should be emphasized that about 4 h elapsed between the ingestion of mercury chloride by the deceased (06:40 a.m.) and the moment of death (10:25a.m.). During this period, the deceased received medical aid which included the administration of fluids and medications, followed by intubation and connection to a ventilator. It should be emphasized that the time to provide effective medical aid was too short, and no further treatment could be implemented, probably due to the large dose of mercury chloride ingested.

If inorganic mercury compounds such as mercury chloride (HgCl_2_) are ingested, the poisoning should be treated in the same way as if a corrosive substance is ingested due to the risk of damage and perforation of the gastrointestinal mucosa. According to the literature data, symptomatic treatment and intensive intravenous therapy are necessary in patients with acute gastrointestinal poisoning. Chelation therapy is also recommended for the treatment of acute intoxication, sometimes combined with plasmapheresis or hemodialysis, especially in patients suffering from renal failure. Chelating agents used in acute poisoning with inorganic mercury include dimercaprol (BAL), d-penicillamine (DPCN), dimercaptopropane sulfonate (DMPS), or succimer (dimercaptosuccinic acid, DMSA) [11–13, 16].

Pathologies detected in the deceased at the autopsy, including dark brown discoloration with superficial necrosis on the line between lips and mucosa, blackish discoloration of the gums at the base of some teeth, very soft esophageal mucosa with flaky exfoliation, whitish hard lesions on the entire stomach wall (thrombotic necrosis) with a gray–white discoloration of the rough mucosa, and very soft duodenal mucosa were also observed by other researchers who reported cases of mercury chloride poisoning [11, 17, 18]. Importantly, mercury levels measured in biological material sampled antemortem and post-mortem in the presented case were lethal and consistent with levels reported for other cases described in the medical literature [17, 19].

## Conclusions

Mercury chloride is still being used in laboratories in Poland. It has an estimated fatal human dose of 1–4 g. This can potentially impair the function of any organ or subcellular structure. HgCl_2_ can produce a range of toxic effects including corrosive injury, severe gastrointestinal disturbances, acute renal failure, circulatory collapse, and eventual death. In the analyzed case, the time taken to provide effective medical aid was short. Unfortunately, no further treatment was implemented. However, a large dose of mercury chloride was ingested by the victim. A description of the analytical procedures for the determination of mercuric chloride in biological materials to be carried out in post-mortem samples has been presented. Additional research is needed to provide analytical methods and a better understanding of how to deal with patients at high HgCl_2_ concentrations.

## Supplementary Information

Below is the link to the electronic supplementary material.

Supplementary file1 (PNG 706 KB)

Supplementary file2 (PNG 687 KB)
