# Supplementary figures and images for: Suicidal intoxication with mercury chloride
Source: Forensic Toxicol. 2022 Dec 24;41(2):304–8. doi: 10.1007/s11419-022-00653-7 (PMC10310567; doi:10.1007/s11419-022-00653-7)

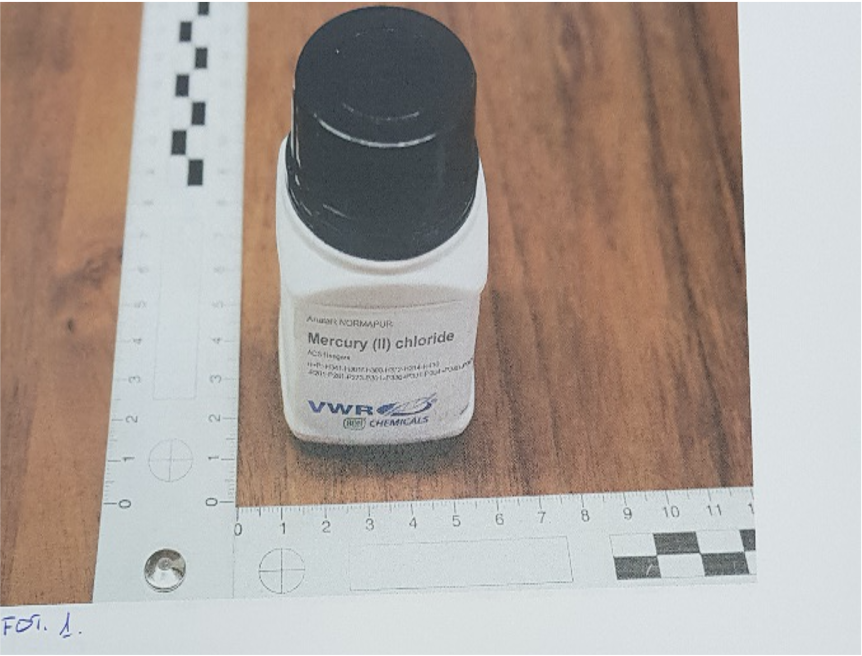

Supplement: Supplementary file 1 — Supplementary file1 (PNG 706 KB) [file 11419_2022_653_MOESM1_ESM.png]

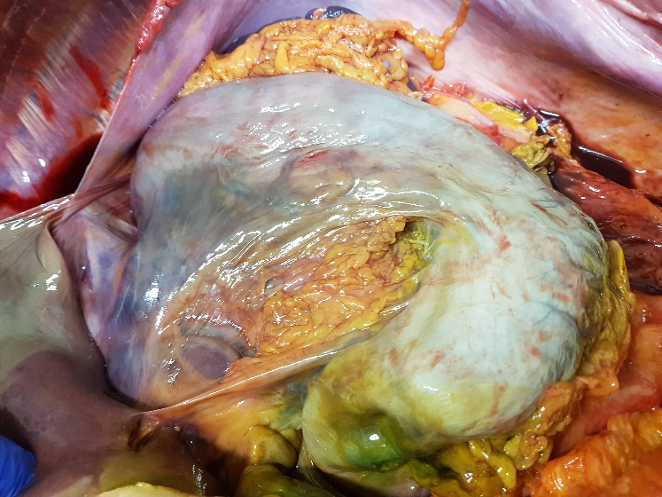

Supplement: Supplementary file 2 — Supplementary file2 (PNG 687 KB) [file 11419_2022_653_MOESM2_ESM.png]
